# Suppression of presbyopia progression with pirenoxine eye drops: experiments on rats and non-blinded, randomized clinical trial of efficacy

**DOI:** 10.1038/s41598-017-07208-6

**Published:** 2017-07-28

**Authors:** Yukari Tsuneyoshi, Akihiro Higuchi, Kazuno Negishi, Kazuo Tsubota

**Affiliations:** 1 Department of Ophthalmology, Keio University School of Medicine, Tokyo, Japan; 2 Institute for Research Promotion, Oita University, Oita, Japan

## Abstract

Various methods can correct presbyopia, but all require devices or surgeries. Recently, supplements or warming devices to relieve presbyopic symptoms have been developed, but no eye drops have been developed. We screened certain compounds possibly related to lens degeneration and identified pirenoxine, which has been used for cataracts, as a possible new pharmacologic treatment for presbyopia. We first researched the anti-presbyopic activity of pirenoxine in rats. The lens elasticity significantly (*p* = 0.028) increased with exposure to tobacco smoke for 12 days, and pirenoxine eye drops significantly (*p* < 0.001) suppressed lens hardening, which causes presbyopia in humans. In a parallel randomized controlled clinical study of the subjects in their fifth decade of life, the objective accommodative amplitude (AA) decreased significantly (*p* < 0.01) by 0.16 diopter (D) in the control group, and there was no detectable change in the treatment group after a 6-month treatment period, suggesting that pirenoxine eye drops might prevent progression of presbyopia. Subjects in their sixth decade of life, in whom the AA was already nearly 0 D, did not show similar results. Pirenoxine eye drops might be a new and the first pharmacologic treatment for preventing progression of presbyopia.

## Introduction

Presbyopia, which is defined as impaired near vision, affects everyone with progressive aging and is nearly universal in people over 55 years of age^1^. One study estimated that there were 1.272 billion cases of presbyopia worldwide in 2011^2^. Furthermore, the global prevalence of presbyopia is predicted to increase to 1.8 billion by 2050^3^.The potential productivity burden of uncorrected or undercorrected presbyopia was estimated to be as much as $25.367 billion or 0.037% of the global gross domestic product in 2011^2^.

Methods to correct presbyopia exist, such as spectacles, multifocal contact lenses, and refractive surgeries. However, each method has its own problems such as the inconvenience of wearing or carrying devices, risk of surgical complications, or decreased quality of vision, and correction of presbyopia without the use of devices or surgeries has not been achieved^4^. Recently, some studies have attempted to prevent progression of presbyopia with supplements^5, 6^ or to recover accommodative ability with periocular warming^7^. We studied the possibility of new pharmacologic treatment for presbyopia.

The limited accommodation in presbyopic eyes is attributed to hardening of the lens with age^8^. We screened some compounds that might be related to lens degeneration and detected pirenoxine as a possible agent (data not shown).

Pirenoxine, a pyridophenoxazine compound resembling xanthommatin, was introduced in 1958 as an eye drop to suppress progression of senile cataracts, and now it is prescribed commonly. Ogino reported that subconjunctival injection of pirenoxine impeded development of cataracts induced by intraperitoneal injection of benzoquinone acetic acid in guinea pigs^9^. He also reported that pirenoxine eye drops prevented the visual acuity (VA) from decreasing in 72 patients with senile cataracts during observation periods ranging from 8 months to 2 years^10^. Until now, no double-blind study has been conducted that sufficiently proved the drug’s efficacy for senile cataract, resulting in the current controversial situation. However, basic research has shown the anti-cataract activity of pirenoxine^11–13^. Oxidative stress, selenite, and calcium ions lead to development of cataract^14–16^. Ciuffi *et al*. reported that pirenoxine prevented lipid peroxidation in guinea-pig lenses^12^. Hu *et al*. reported that pirenoxine prohibited lens protein turbidity induced by UVC, selenite, and calcium^13^. Liao *et al*. reported that pirenoxine binds up to six selenite anions and possesses ditopic recognition properties, which is a rationale for pirenoxine as a cataract therapy^11^.

In the current study, we first investigated the anti-presbyopic activity of pirenoxine in rats and then studied the efficacy of pirenoxine eye drops in association with the accommodative amplitude (AA) and functional VA (FVA)^17^ in a randomized controlled trial.

## Results

### Rats Experiments

#### Establishment of the presbyopia rat model

**Figure 1:**
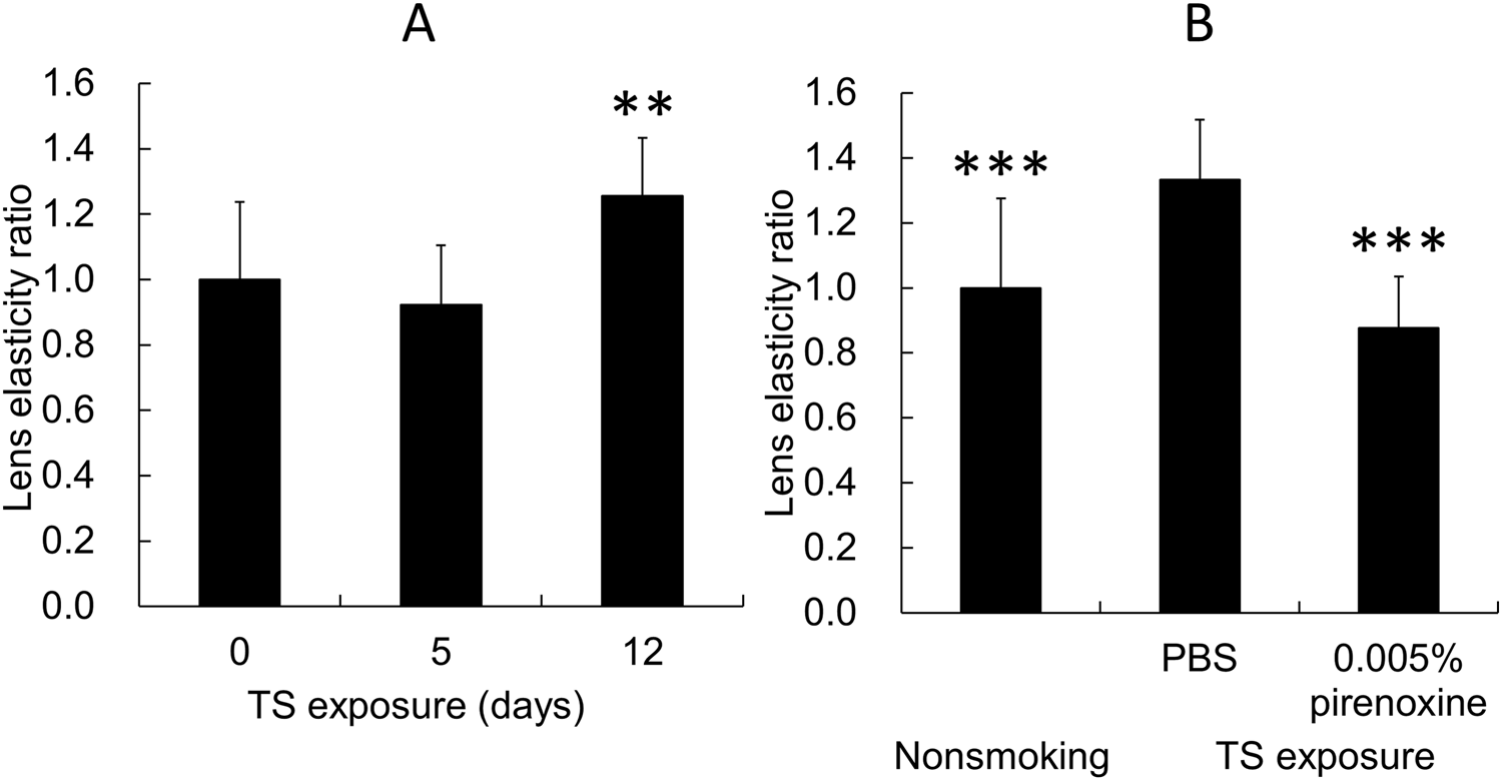
Effect of pirenoxine eye drops on lens elasticity. (**A**) The effect of exposure to TS on the elasticity of rat lenses. The horizontal axis shows the period of exposure to TS, i.e., 0, 5, and 12 days (n = 8, 5, and 10, respectively). The data are expressed as the ratio to the mean in the 0-day treatment group. **Indicates a significant (*p* = 0.028) difference from the result in the 0-day treatment. (**B**) Evaluation of the elasticity ratio of the rat lenses exposed to TS treated with 0.005% pirenoxine or PBS (n = 14 and 11, respectively). The data are expressed as the ratio to the mean in the nonsmoking group. ***Indicates significant (*p* = 0.001 for the nonsmoking group (n = 12) and *p* < 0.001 for the pirenoxine treatment group) differences from the results with PBS treatment in the nonsmoking group and the pirenoxine group. The results are expressed as the mean ± standard deviation.

Figure 1A shows the effect of tobacco smoke (TS) on the lens elasticity. The data are expressed as the ratio to the mean in the 0-day treatment group. The lens elasticity significantly (*p* = 0.028) increased by exposure to TS for 12 days.

#### Assessment of the efficacy of pirenoxine in rats

Figure 1B shows the efficacy of pirenoxine treatment in rats. The data are expressed as the ratio to the mean in the nonsmoking group. The lens elasticity was significantly (*p* < 0.001) lower in the pirenoxine group compared to the PBS group.

#### Clinical assessment of pirenoxine

No treatment-related complications developed during treatment with pirenoxine and artificial tears. Table 1 shows the baseline characteristics of the study subjects. There was no significant difference in the key characteristics between the treatment group (TG) and control group (CG).

**Table 1:** The baseline characteristics of subjects.

	Total (n = 18)	TG (n = 10)	CG (n = 8)	*p* value
Age (years)	49.2 ± 3.1	48.7 ± 3.4	49.8 ± 2.9	0.50
Subjective refraction (SE) (D)	−1.44 ± 1.22	−1.2 ± 0.77	−1.75 ± 1.62	0.36
CDVA (logMAR)	−0.18 ± 0.09	−0.20 ± 0.07	−0.16 ± 0.11	0.36
DCNVA (logMAR)	0.49 ± 0.26	0.49 ± 0.26	0.49 ± 0.29	0.98
Objective AA (D)	0.61 ± 0.49	0.61 ± 0.56	0.61 ± 0.44	1.00

The data are expressed as the mean ± standard deviation.

SE = spherical equivalent; D = diopters; logMAR = logarithm of the minimum angle of resolution; CDVA = corrected distance visual acuity; DCNVA = distance corrected near visual acuity; AA = accommodative amplitude.

#### Effect on AA

**Figure 2:**
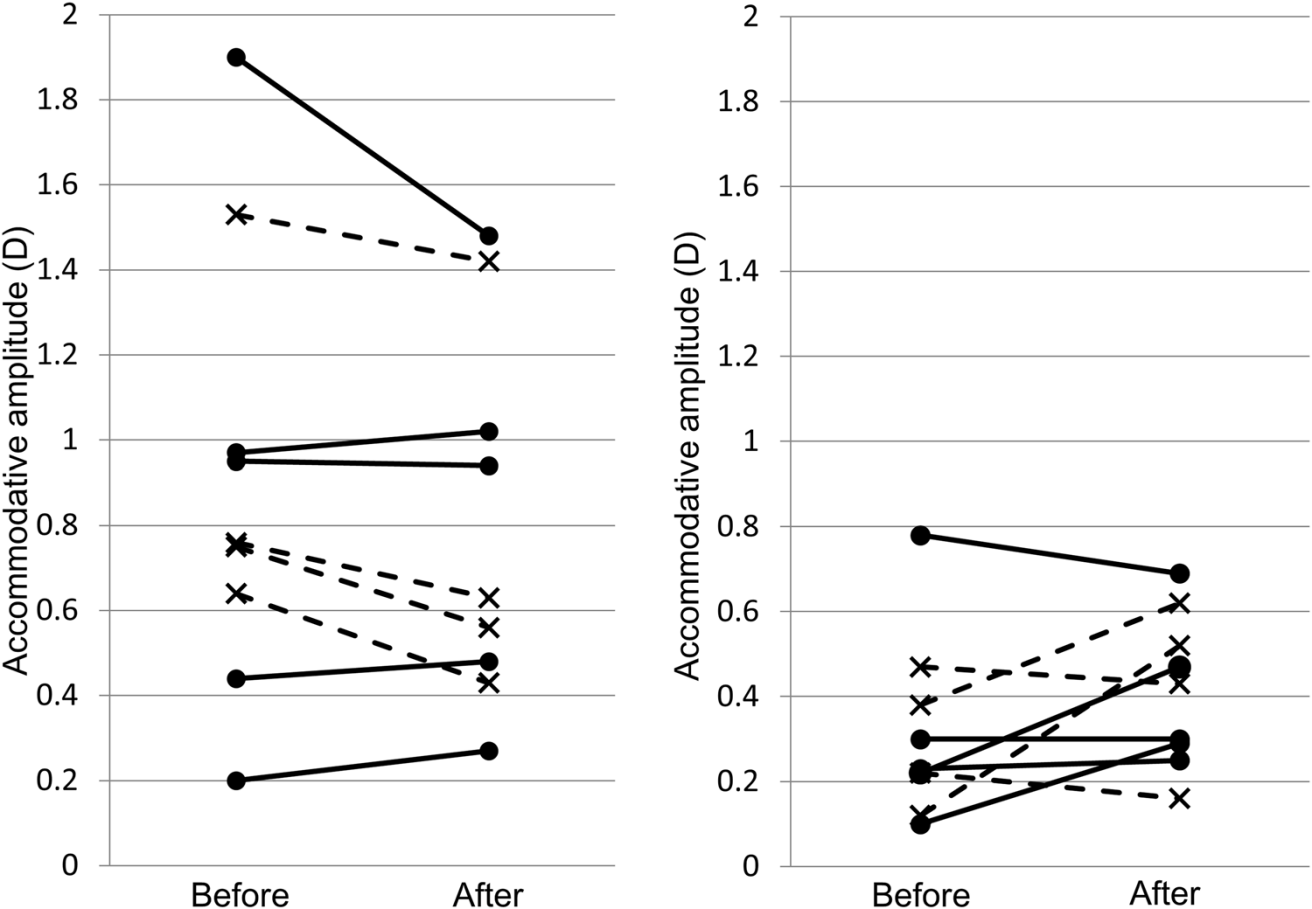
Accommodative amplitude before and after treatment in respective subjects. (Left) Subjects in their fifth decade of life; (right) subjects in their sixth decade of life. The solid lines and circles represent the treatment group; the dashed lines and crosses represent the control group.

The mean objective AAs before and after treatment were 0.61 ± 0.56 D and 0.62 ± 0.41 D in the TG and 0.61 ± 0.44 D and 0.60 ± 0.37 D in the CG; there were no significant (*p* = 0.86 and *p* = 0.88, respectively) changes in either group. However, when assessed separately by age bracket, a difference was seen between the TG and CG (Table 2). For subjects in their fifth decade of life, the objective AAs in the CG decreased significantly (*p* < 0.01) by 0.16 ± 0.05, whereas there was no significant (*p* = 0.59) change in the TG. For subjects in their sixth decade of life, the changes in the CG and TG did not differ significantly (*p* = 0.31 for both comparisons). The changes in each subject are shown in Fig. 2.

**Table 2:** Changes in the objective accommodative amplitude by age bracket.

		Before treatment (D)	After treatment (D)	Difference (D) (after-before)	*p* value
40s	TG (n = 5)	0.89 ± 0.65	0.84 ± 0.48	−0.05 ± 0.21	0.59
	CG (n = 4)	0.92 ± 0.41	0.76 ± 0.45	−0.16 ± 0.05	<0.01*
50s	TG (n = 5)	0.33 ± 0.26	0.40 ± 0.18	0.07 ± 0.14	0.31
	CG (n = 4)	0.30 ± 0.16	0.43 ± 0.20	0.14 ± 0.22	0.31

The data are expressed as the mean ± standard deviation.

40s = fifth decade of life; 50s = sixth decade of life.

*Significant correlation (*p* < 0.05 with the Bonferroni correction).

#### Effect on other parameters

The spherical equivalent (SE) of the subjective refractions, distance corrected near visual acuity (DCNVA), and minimal pupillary diameter did not change significantly in any group (Table 3). Table 4 shows the changes in the distance FVA (DFVA) and distance corrected near FVA (DCNFVA) in the TG and CG based on age. The DFVA did not change significantly in any group. Regarding the DCNFVA, the patients in their sixth decade of life in the TG improved significantly (*p* < 0.01) after treatment, and no significant changes were seen in the other groups.

**Table 3:** Changes in the SE of the objective refraction, DCNVA, and minimal pupillary diameter.

		Subjective refraction (SE) (D)			DCNVA (logMAR)			Minimal pupillary diameter (mm)		
		Before treatment	After treatment	*p* value	Before treatment	After treatment	*p* value	Before treatment	After treatment	*p* value
40s	TG (n = 5)	−0.85 ± 0.68	−0.65 ± 0.77	0.23	0.40 ± 0.33	0.22 ± 0.16	0.14	3.92 ± 1.58	3.68 ± 1.08	0.44
	CG (n = 4)	−1.69 ± 1.36	−1.56 ± 1.21	0.50	0.32 ± 0.22	0.28 ± 0.25	0.53	4.05 ± 0.79	3.85 ± 0.67	0.54
50s	TG (n = 5)	−1.55 ± 0.76	−1.30 ± 0.68	0.13	0.58 ± 0.13	0.48 ± 0.29	0.41	3.82 ± 1.65	3.92 ± 1.50	0.61
	CG (n = 4)	−1.81 ± 2.07	−1.78 ± 2.19	0.85	0.65 ± 0.27	0.70 ± 0.22	0.59	3.60 ± 0.44	3.90 ± 0.59	0.25

The data are expressed as the mean ± the standard deviation.

SE = spherical equivalent; D = diopters; logMAR = logarithm of the minimum angle of resolution; DCNVA = distance corrected near visual acuity; TG = treatment group; CG = control group; 40s = fifth decade of life; 50s = sixth decade of life.

**Table 4:** Changes in the DFVA and DCNFVA.

		DFVA (logMAR)			DCNFVA (logMAR)		
		Before treatment	After treatment	*p* value	Before treatment	After treatment	*p* value
40s	TG (n = 5)	−0.15 ± 0.03	−0.09 ± 0.07	0.23	0.56 ± 0.42	0.31 ± 0.23	0.20
	CG (n = 4)	−0.09 ± 0.05	−0.10 ± 0.03	0.79	0.50 ± 0.41	0.54 ± 0.29	0.76
50s	TG (n = 5)	−0.09 ± 0.08	−0.11 ± 0.04	0.49	0.86 ± 0.13	0.65 ± 0.19	<0.01*
	CG (n = 4)	−0.02 ± 0.17	−0.02 ± 0.17	0.39	0.90 ± 0.13	0.76 ± 0.32	0.22

The data are expressed as the mean ± standard deviation.

*Significant correlation (*p* < 0.05 with the Bonferroni correction).

DFVA = distance functional visual acuity; DCFVA = distance corrected near functional visual acuity; logMAR = logarithm of the minimum angle of resolution; TG = treatment group; CG = control group; 40s = fifth decade of life; 50s = sixth decade of life.

## Discussion

The current study is the first to investigate the efficacy of pirenoxine eye drops for presbyopia. In the animal experiments, we used the exposure to TS method because it has been reported that the AA of smokers was significantly lower than that of nonsmokers^18^. The lens elasticity increased significantly because of exposure to TS for 12 days. This result indicated that exposure to TS induced lens hardening, which causes presbyopia in humans^16, 17^, and that the rats exposed to TS can be used as a presbyopia model. We assessed the efficacy of pirenoxine using the model rat subsequently. The results showed that the pirenoxine eye drops significantly suppressed hardening of the lens caused by exposure to TS. This suggested that pirenoxine eye drops might prevent presbyopia. Therefore, we conducted the clinical randomized controlled study.

In the clinical study, when assessed separately by age, the objective AA decreased significantly by 0.16 D in the CG in the subjects in their fifth decade of life, while there was no detectable change in the AA in the TG. There was no significant change in the objective AA in subjects in their sixth decade of life in the CG and TG. Anderson and Stuebing assessed the objective AA as a function of age and showed that it decreased dramatically from 35 to 39 years and reached a plateau during the sixth decade of life, with almost no or very little decline between 50 to 60 years of age^18^. Considering their results, the absence of a detectable change in the objective AA in the TG in subjects in their fifth decade of life suggested that pirenoxine eye drops might prevent the decline in the objective AA, i.e., the progression of presbyopia. Similar results might not be seen among subjects in their sixth decade of life because the objective AA was already nearly zero before treatment. These results suggest that pirenoxine eye drop might prevent progression of presbyopia.

The ARK-1s measures the refraction by analyzing 1- to 3.5-mm pupillary zones with a charge-coupled device. When the pupillary diameter is smaller than 3.5 mm, unmeasurable data are treated as missing data. The objective AA has been reported to be affected by the pupillary size if the measurement method is dependent on spherical aberrations^19^. Therefore, if there is a difference in the measurable pupillary zone between before and after treatment, the measured objective AA data might be affected. In the current study, there was no significant difference in the minimal pupillary diameter between before and after treatment, so the observed difference in the objective AA is considered to not be affected by pupillary constriction.

Katada *et al*., who assessed the relationship between near visual function and subjective AA^17^, found that the DCNVA and DCNFVA were correlated significantly with the subjective AA and the slope of the linear regression between the DCNFVA and AA is steeper than between the DCNVA and AA. In the current study, a change in the AA was detected only in the CG in subjects in their fifth decade of life, i.e., a decline of 0.16 D. However, there was no significant change in the DCNVA or DCNFVA reflecting the decline in the AA. This might have been because a decline of 0.16 D was small and no effect on the near visual function was detected. The DCNVA did not change significantly in any other groups, but the DCNFVA improved significantly in the TG in subjects in their sixth decade of life, although the AA did not change significantly. This suggests that some mechanism other than an improvement in the AA might induce an improvement in the DCNFVA. Pirenoxine eye drops have been reported to reduce the optical density in the cortical lens layers and beneath the posterior capsule in humans^20^. It also has been reported that higher lens density worsens the contrast sensitivity^21^ and the near visual function of presbyopes improves by increasing the contrast^22^. Considering that the lens density increases with age^23^, improvement in the lens transparency might be a reason for the improved DCNFVA in the TG in subjects in their sixth decade of life.

The current study had some limitations. The small number of subjects was related to its exploratory nature and the difficulty in enrolling a large number of subjects. There might have been more effects that could not be detected as a result of the small sample size. Another study with more subjects should be performed. We used 0.005% pirenoxine eye drops because the safety of that dose has already been established as a drug for cataract, but we did not assess the efficacy of other concentrations. Therefore, a higher concentration might have resulted in greater improvement. In addition, the efficacy of pirenoxine was proved by the change in the AA, but an effect on the subjective measurement was not detected. It is thought that the natural progression of presbyopia is not so drastic that the DCNVA or DCNFVA does not decrease significantly over a 6-month period. A longer study might be necessary to determine the subjective effects.

In conclusion, 0.005% pirenoxine eye drops, which have been used for cataracts, might be promising as a new and the first pharmacologic treatment for presbyopia.

## Methods

### Rat experiments

The Keio University Institutional Animal Care and Use Committee approved all animal experiments (approval number: 09079), which were performed in Keio University School of Medicine, according to the Institutional Guidelines on Animal Experimentation at Keio University. Six-week-old male Sprague-Dawley rats were purchased from CLEA Japan, Inc. (Tokyo, Japan).

### Establishment of a presbyopia rat model

The TS exposure rat model was prepared as described previously^24, 25^. Briefly, rats were placed in an experimental smoking chamber (60 × 40 × 35 cm) with continuous fresh air ventilation for 3 hours daily for 12 days. Three hundred milliliters of mainstream cigarette smoke prepared from cigarettes containing 14 mg of tar (Seven Stars, Japan Tobacco, Tokyo, Japan) was injected into the smoking chamber every 30 min for a total of six times over a 3-hour period daily for 0, 5, or 12 days (n = 8, 5, or 10, respectively) during the exposure period. After the exposure period, the lenses were collected under anesthesia to measure the lens elasticity using a height gauge and electronic balance (Supplemental Fig. 1). The lenses were placed on the electronic balance and the tip of the height gauge was put in contact with the lens (Supplemental Fig. 2A). After adjusting to zero, the tip of the height gauge was turned down to apply pressure on the lens (Supplemental Fig. 2B). The lens elasticity was calculated by dividing the weight change by the amount of movement of the tip of the height gauge.

### Assessment of the efficacy of pirenoxine in rats

The rats were divided into three groups, i.e., the nonsmoking group (normal group not exposed to TS), the phosphate-buffered saline (PBS) group (exposure to TS treated with PBS eye drops), and the pirenoxine group (exposure to TS treated with pirenoxine eye drops) (Kary Uni 0.005%, Santen Pharmaceutical Co., Ltd., Osaka, Japan). The treatment with eye drops was started on the first day of the exposure to TS. Five microliters of each eye drop was administered 4 times daily. Each day, the first eye drop was applied before the exposure to TS, and the subsequent eye drops were applied after the exposure to TS. The lens elasticity was measured after 12 days of exposure to TS and eye drop treatment.

### Clinical study

The Institutional Review Board for Human Studies of Keio University School of Medicine approved the prospective parallel randomized controlled clinical study, which followed the tenets of the Declaration of Helsinki. The protocol was registered with the UMIN Clinical Trials Registry (UMIN000019050) on September 17, 2015. Healthy volunteers were recruited from October 1, 2015 to January 14, 2016. The inclusion criteria were an age of 40 to 54 years, male gender, 20/20 or better best-corrected VA, no history of refractive surgery, and no ophthalmic diseases other than refractive errors. All subjects provided informed consent before the examinations. In a previous study on the effect of periocular warming on accommodative amplitude^7^, the changes in accommodative amplitude were detected more sharply in men. Because of this, we planned to include only men to detect the effect with the smaller sample size. This study was purely exploratory in nature and no preceding study has been performed regarding calculation of the effect size. Therefore, the sample size was determined considering the practical number of volunteers we could collect and reasonable time to spend on the examinations. Nineteen right eyes of 19 healthy volunteers were enrolled in this study at Keio University Hospital. The subjects were assigned randomly to either the TG or the CG following simple randomization procedures using a computer-generated list of random numbers. The allocation ratio was 1:1. One subject assigned to the CG was excluded from the analysis because the primary outcome (objective AA) was unmeasurable due to the small pupil size. There were no other loses and exclusions. Therefore, the number of eyes included in the analysis was 18. The mean age of the participants was 49.2 ± 3.15 years (range, 44–54 years). The TG (n = 9) received pirenoxine eye drops, and the CG (n = 9) received artificial tears (Soft Santear, Santen Pharmaceutical Co., Ltd.). The subjects were instructed to instill the eye drops 4 times daily for 6 months. The interventions started between January 20 and February 24, 2016 and ended between July 21 and August 25 2016. The primary outcome was objective AA and the secondary outcomes were DCNVA and FVA. Data were measured before and after interventions.

### Refractive and VA measurements

The distance subjective refractions and VA were measured using a space saving chart (SSC-350, Nidek Co. Ltd., Aichi, Japan). The near VA was measured using the Landolt C Near Card (Good-Lite Co., Elgin, IL, USA) at 40 cm. The subjective refractive data were entered into a spreadsheet in negative cylindrical form, and the spherical equivalent (SE) of each refraction was determined. One experienced examiner (S.M.) measured the subjective refractions and VA.

### AA and pupillary diameter

The objective AA was measured using the Nidek Auto Ref/Keratometer ARK-1s (Nidek Co. Ltd.), which is an Auto Ref/Keratometer equipped with an accommodation measurement system. The objective refraction and the pupillary diameter were measured continuously while the patients focused on the internal target that moves from distance to near. Astigmatism was corrected with built-in cylinder lenses during the measurement. The objective AA was calculated as the difference in the refraction between the mean value obtained under the fogging lens and the value obtained at the near point of accommodation. The minimal pupillary diameter during the measurement also was determined.

### FVA measurement

The FVA is the average of the VAs measured continuously during a certain time frame, which represents the VA in daily life more efficiently than the usual VA^17, 24, 25^. The FVA measurement has been reported to detect early presbyopia more sensitively than the usual VA measurement^17^. The AS-28 (Kowa Co. Ltd., Aichi, Japan) was used to measure the FVA. The measurement time frame was set to 60 seconds. The DFVA was measured with the best correction for distance. The DCNFVA was measured with −2.50 D added to the best distance correction because the AS-28 only presents far VA charts. Therefore, the DCNFVA represents the average of the DCNVA at 40 cm for 60 seconds.

### Literature search

We conducted a PubMed literature search up to January 2017 using the keywords presbyopia, accommodation, and pirenoxine to search for similar studies.

### Statistical analysis

The values are expressed as the means ± standard deviations. A probability of less than 5% (*p* < 0.05) was considered significant. For the animal experiments, Dunnett’s test was used to determine the significance of differences. For the clinical study, the values from the two study groups were compared using the Student t-test. The values before and after treatment were compared using the paired t-test. The Bonferroni correction was used for multiple tests. Statistical analyses were performed using SPSS version 23 for Windows software (IBM Corp, Armonk, NY).

## Electronic supplementary material

Supplementary Materials
