# Author Correction: Suppression of presbyopia progression with pirenoxine eye drops: experiments on rats and non-blinded, randomized clinical trial of efficacy

**DOI:** 10.1038/s41598-020-62436-7

**Published:** 2020-04-16

**Authors:** Yukari Tsuneyoshi, Akihiro Higuchi, Kazuno Negishi, Kazuo Tsubota

**Affiliations:** 1 Department of Ophthalmology, Keio University School of Medicine, Tokyo, Japan; 2 Institute for Research Promotion, Oita University, Oita, Japan

Correction to: *Scientific Reports* 10.1038/s41598-017-07208-6, published online 28 July 2017

The Article contains errors in the Reference list. The authors omitted the below papers, which are listed as References 1–5. These should be cited in the discussion section as below:

“This result indicated that exposure to TS induced lens hardening, which causes presbyopia in humans^16, 17^, and that the rats exposed to TS can be used as a presbyopia model.”

should read:

“This result indicated that exposure to TS induced lens hardening, which causes presbyopia in humans^1,2^, and that the rats exposed to TS can be used as a presbyopia model.”

And,

“Anderson and Stuebing assessed the objective AA as a function of age and showed that it decreased dramatically from 35 to 39 years and reached a plateau during the sixth decade of life, with almost no or very little decline between 50 to 60 years of age^18^.”

should read:

“Anderson and Stuebing assessed the objective AA as a function of age and showed that it decreased dramatically from 35 to 39 years and reached a plateau during the sixth decade of life, with almost no or very little decline between 50 to 60 years of age^3^.”

Finally, in the Methods section under subheading ‘Establishment of a presbyopia rat model’,

“The TS exposure rat model was prepared as described previously^24, 25^.”

should read:

“The TS exposure rat model was prepared as described previously^4,5^.”
