# Supplementary material for: Suppression of presbyopia progression with pirenoxine eye drops: experiments on rats and non-blinded, randomized clinical trial of efficacy
Source: Sci Rep. 2017 Jul 28;7:6819. doi: 10.1038/s41598-017-07208-6 (PMC5533700; doi:10.1038/s41598-017-07208-6)
Supplement: Supplementary file 1 — Supplementary Materials [file 41598_2017_7208_MOESM1_ESM.pdf]

Supplementary Materials for

Suppression of presbyopia progression with pirenoxine eye drops: experiments on rats and non-blinded, randomized clinical trial of efficacy

Yukari Tsuneyoshi1, Akihiro Higuchi, Kazuno Negishi and Kazuo Tsubota

This PDF file includes:

Figs. S1 and S2

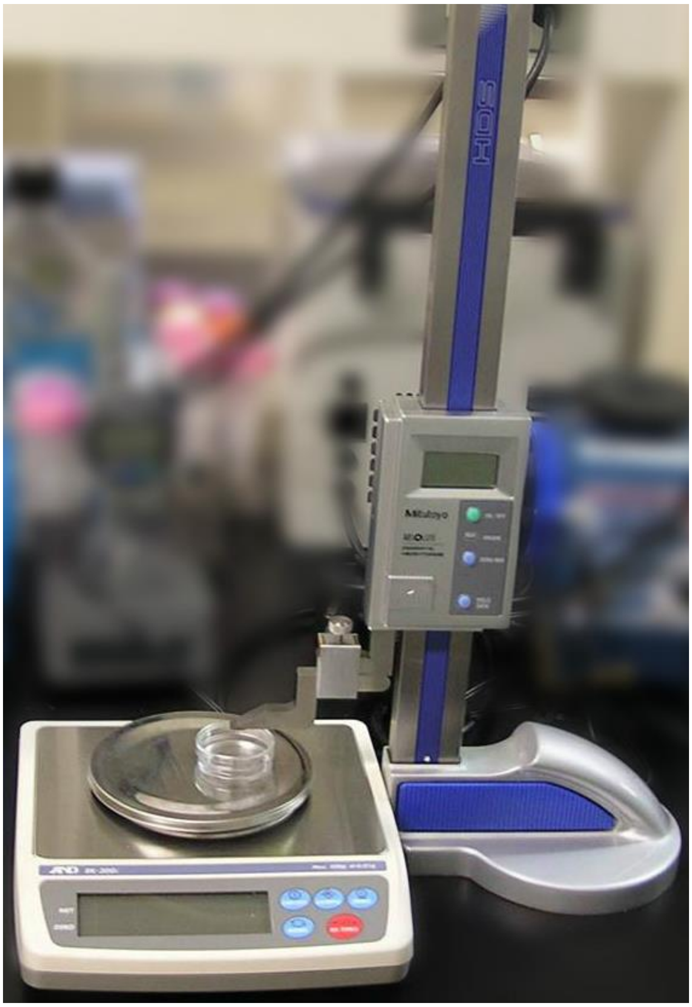

Fig S1. The Setting for Lens Elasticity Measurement

The lens elasticity was measured using a height gauge and electronic balance.

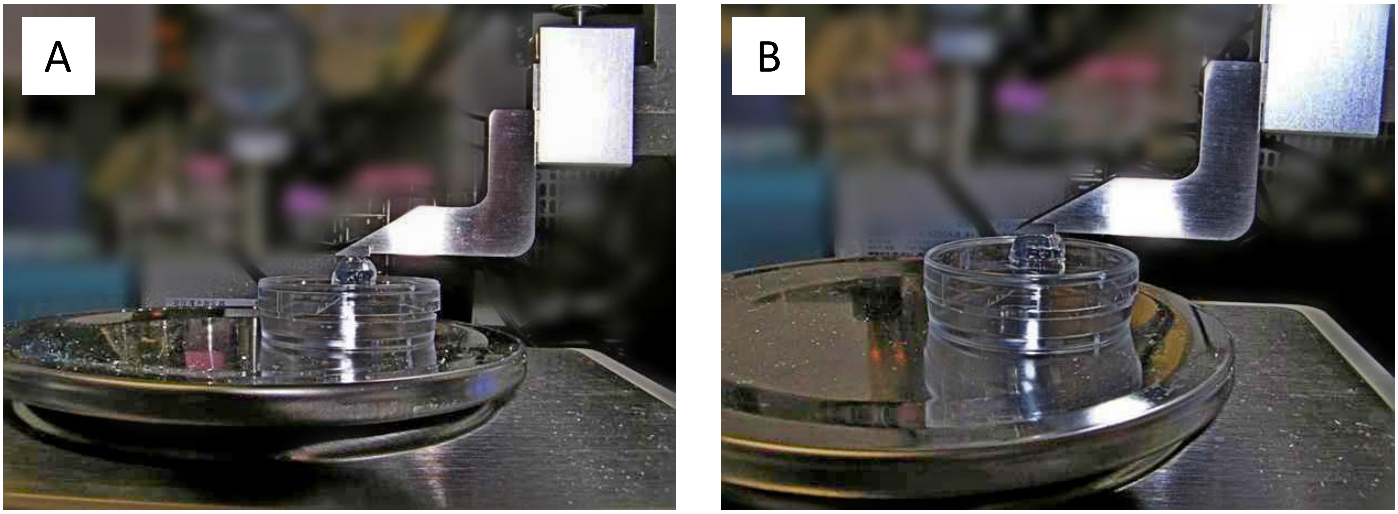

Fig S2. The procedures for lens elasticity measurement
Photos are examples with pig lenses.
(A) The lenses were placed on the electronic balance and the tip of the height gauge was put in contact with the lens. (B) After adjusting to zero, the tip of the height gauge was turned down to apply pressure on the lens.
